# Machine Learning Models of Phase Contrast Images Predict Efficiency of Human Pluripotent Stem Cell Differentiation to Cardiomyocytes

**DOI:** 10.1002/bit.70241

**Published:** 2026-05-14

**Authors:** Austin K. Feeney, Yaniv Ovadia, Aaron D. Simmons, Christian Elabd, Claire J. Peplinski, Frank Li, Sean P. Palecek

**Affiliations:** 1 Department of Biomedical Engineering, University of Wisconsin-Madison, Madison, Wisconsin, USA; 2 Medical Scientist Training Program, University of Wisconsin School of Medicine and Public Health, Madison, Wisconsin, USA; 3 Stately Bio Inc., Palo Alto, California, USA; 4 Department of Chemical and Biological Engineering, University of Wisconsin-Madison, Madison, Wisconsin, USA

**Keywords:** cardiomyocyte, differentiation, human pluripotent stem cell, machine learning, phase contrast imaging

## Abstract

Terminal cell types derived from human pluripotent stem cells (hPSCs) are at the forefront of emerging cell and gene therapy products. hPSC-derived cardiomyocytes (hPSC-CMs) are of particular interest in understanding and treating heart disease, which is highly prevalent worldwide; however, hPSC-CM manufacturing robustness is a roadblock to these applications. Non-destructive methods to monitor hPSC-CMs and predict differentiation efficiency throughout the process are needed. Here, we demonstrate a supervised machine learning approach utilizing random projection-based feature embedding and Linear Discriminant Analysis that can predict Day 12 CM purity from phase contrast images as early as 1 day after initiation of differentiation. In contrast, a heuristic based on a live-cell cTnT-GFP reporter provided limited information until Days 7–9. In summary, we provide evidence that machine learning analysis of phase contrast images is a promising approach for predicting hPSC-CM differentiation efficiency during early differentiation stages. Future efforts could use these models to inform the improvement of CM differentiation protocols and support CM biomanufacturing.

## 1 | Introduction

Human pluripotent stem cells (hPSCs) possess substantial promise for modeling and treating human diseases. Following the isolation of hPSCs in 1998, protocols to differentiate hPSCs towards diverse terminal cell types have been of worldwide interest, with 115 clinical trials for 83 hPSC-derived cell types by the end of 2024 (Kirkeby et al. 2025; Thomson et al. 1998). Given the ability of hPSCs to differentiate into any cell type in the body, reconstructing the dynamic in vivo milieu for proper cell-fate acquisition in vitro remains challenging.

Despite medical advances, heart disease has been the leading cause of death in the United States since 1921 (CDC). Protocols to generate cardiomyocytes from hPSCs (hPSC-CMs) have the capacity to revolutionize heart disease management but exhibit high cell-line and batch variability (Laco et al. 2018). Thus, methods to monitor and forecast hPSC-CM differentiation efficiency will be essential to improve hPSC-CM manufacturing for clinical translation. Most existing strategies to monitor CM differentiation progression are low-throughput and destructive. Ideally, next-generation strategies to track CM differentiation would occur in real time, integrate with culture equipment, and predict CM differentiation efficiency during early process stages.

hPSCs undergo dramatic morphological changes during CM differentiation (Ren et al. 2021). hPSCs are round, tightly packed, colony-forming cells with a high nucleus to cytoplasm ratio (Kato et al. 2016). In contrast, CMs are rod-shaped cells with specialized structures that influence morphology, such as gap junctions, densely packed mitochondria, and organized contractile sarcomeres (Walker and Spinale 1999). Unfortunately, progenitor stage morphology changes during hPSC-CM differentiation are poorly characterized. Yet, one can visually recognize when a CM differentiation batch may be successful prior to spontaneous contraction, a hallmark of hPSC-CM function. However, these assessments are imprecise, practitioner-dependent, and difficult to correlate with the true percentage of CMs by flow cytometry or immunocytochemistry (ICC). Currently, real-time, non-invasive assessment of CM differentiation outcomes relies on hPSCs engineered to fluorescently report cTnT, a known CM identity marker (Waas et al. 2019).

In recent years, machine learning analysis of label-free, phase contrast images has shown great promise in capturing biological signals (Christiansen et al. 2018; Ounkomol et al. 2018). The objective of this study was to predict hPSC-CM differentiation efficiency from early morphological signals captured by phase contrast images. We hypothesized that morphology changes reflect cell identity transitions and that machine learning models could predict hPSC-CM differentiation outcome from phase contrast images. Phase contrast images were collected from Days 0 to 12, and hPSC-CM differentiation efficiency was quantified from Day 12 cTnT ICC based on the cTnT+ area. Temporal sequences of images were encoded as lower-dimensional embeddings and used to train a Linear Discriminant Analysis (LDA) supervised machine learning model to assign predicted differentiation efficiency scores, which were compared with Day 12 ground-truth differentiation efficiencies. Phase contrast predictions were also compared to fluorescent signals from a cTnT-GFP live-cell reporter during differentiation. Importantly, the random projection LDA machine learning approach offered several advantages over more complex approaches for our phase contrast imaging data set, including high computational efficiency, low overfitting risk with sample size constraints, and an interpretable linear decision boundary. Herein, we demonstrate a label-free, non-invasive approach to monitor hPSC-CM manufacturing outcome, which can be used to optimize differentiation efficiency, reduce variability, and accelerate the clinical translation of hPSC-CMs for heart disease treatment.

## 2 | Results and Discussion

**FIGURE 1 |:**
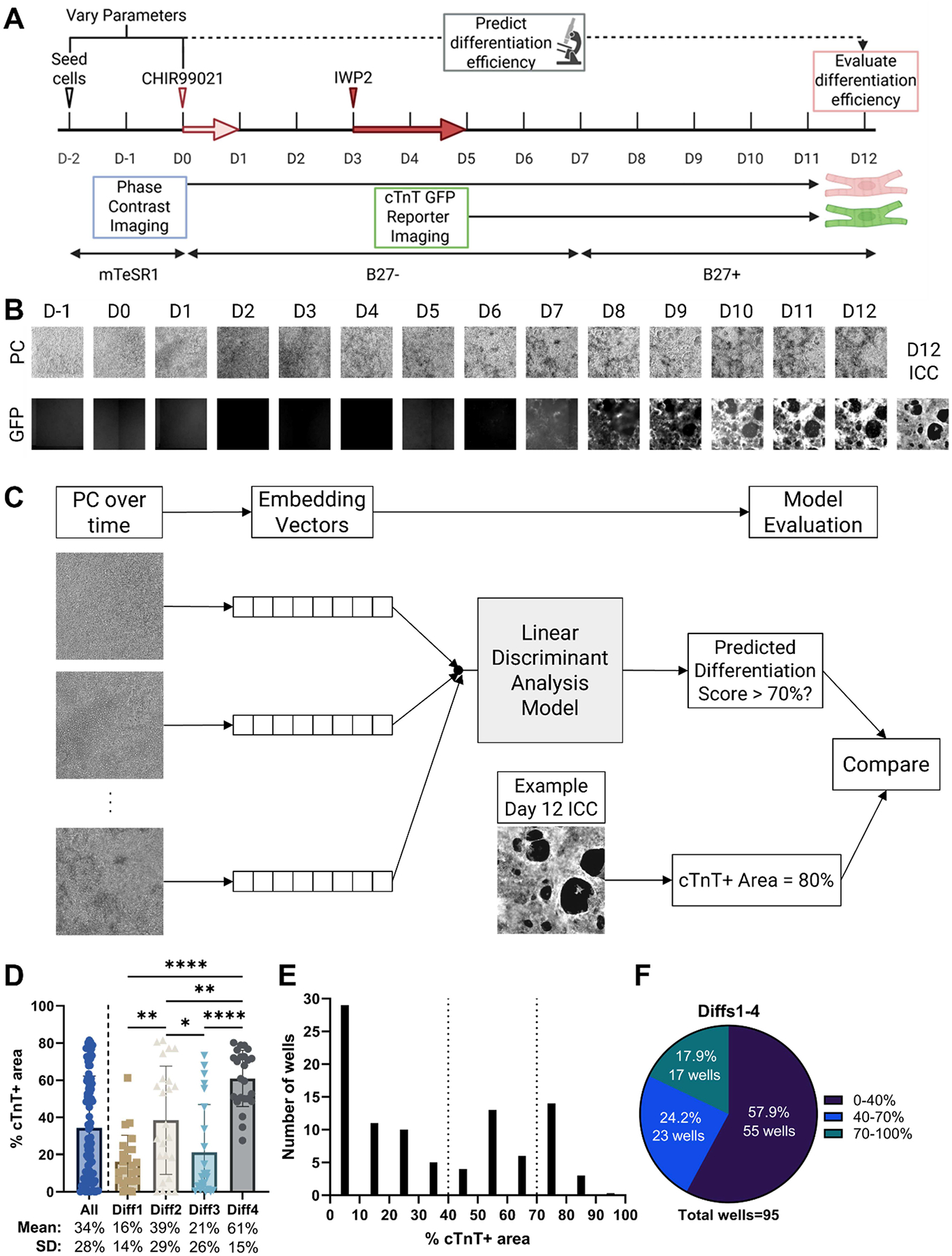
Overview of hPSC-CM differentiation design and results, imaging strategy, and modeling strategy. (A) Schematic of the hPSC-CM differentiation protocol, the parameter screen strategy used to obtain differentiation variability, and the imaging strategies used to monitor hPSC-CM differentiation efficiency. (B) Representative images from Day −1 to Day 12 for phase contrast (PC) and the cTnT-GFP reporter (GFP) fluorescence used to predict terminal hPSC-CM differentiation efficiency on Day 12 from immunocytochemistry (ICC) for cTnT. (C) Schematic of modeling approach using temporal phase contrast images to encode embedding vectors, which serve as the input for an LDA model. The model yields a predicted differentiation efficiency score, which is compared against ground truth % cTnT+ area at Day 12 (range 0%–100%). (D) Percentage of CMs by area (% cTnT+ area) from Day 12 ICC binarization across differentiation experiments. Points represent independent differentiation wells, and bars display mean ± SD. *N* = 95 wells (all Diffs). *N* = 24 wells (Diffs 1, 2, 4). *N* = 23 wells (Diff 3). *p*-values from one-way ANOVA with Tukey's post hoc test (excluding the all column separated by a vertical dotted line). *p < 0.05, **p < 0.01, ***p < 0.001, and ****p < 0.0001. (E) Histogram for hPSC-CM differentiation efficiency (percentage of cTnT+ cells by area) across all four differentiation experiments. *N* = 95 wells. Vertical dotted lines at 40% and 70% cTnT+ cells indicate thresholds for low and high efficiency CM differentiation, respectively. (F) Pie chart for high (> 70% cTnT+ cells), low (< 40% cTnT+ cells), and intermediate (40%–70% cTnT+ cells) differentiation efficiency bins across differentiations. Diff, differentiation.

To differentiate hPSC-CMs, we used the GiWi (GSK3β inhibitor, Wnt inhibitor) protocol (Lian et al. 2013), which temporally activates then inhibits Wnt signaling with CHIR99021 and IWP2 (Figure 1A). Because the differentiation outcome is drastically influenced by cell density and CHIR99021 concentration (Laco et al. 2018), we varied these parameters to induce variability in process outcome and evaluate if machine learning models trained on phase contrast images acquired early in differentiation could predict differentiation efficiency (Figures 1A and S1A–F). Differentiation efficiency (% cTnT+ area) was quantified on Day 12 by creating binary masks from cTnT ICC images (Figure 1B). Differentiations were performed in an hPSC line engineered to co-express GFP and cTnT (Wrighton et al. 2014). Phase contrast and cTnT-GFP live-cell fluorescence images were acquired from Days 0–12 and Days 5–12, respectively (Figure 1B).

To predict Day 12 CM differentiation efficiency, we encoded sequences of images as lower-dimensional embedding vectors and trained a multiclass LDA model to compute a score for differentiation efficiency (Figure 1C). The random projection LDA approach offered statistical and computational efficiency while minimizing overfitting risk compared to more complex approaches. We evaluated model performance using standard classification metrics under multiple cross-validation strategies (Figure 1C). Phase contrast models were also compared to the performance of a heuristic based on the median fluorescence intensity of a cTnT-GFP live-cell reporter (Figure 1B). In total, four independent differentiations were performed, and differentiation efficiency, or the percentage of CMs, was quantified from the cTnT+ area from Day 12 cTnT ICC images. Differentiation outcomes were classified as high efficiency (> 70% CMs) or low efficiency (< 40% CMs) for modeling prediction efforts. Across experiments, differentiation efficiency ranged from 0% to 81.5% (mean = 34.4%; Figure 1D), with 17.9% high (> 70% CMs) and 24.2% low (< 40% CMs) CM purity wells (Figure 1E). Differentiation 1 efficiency ranged from 0% to 61.3% (mean = 16.2%; Figure 1D), with 0% high and 95.8% low CM purity wells (Figure S1B). Differentiation 2 efficiency ranged from 0.04% to 81.5% (mean = 38.5%; Figure 1D), with 20.8% high and 54.2% low CM purity wells (Figure S1D). Differentiation 3 was designed for more variability with additional CHIR99021 concentrations (Figure S1E,F), and efficiency ranged from 0% to 73.3% (mean = 21.3%; Figure 1D), with 4.3% high and 73.9% low CM purity wells (Figure S1F). Differentiation 4 was designed to incorporate handling variability with 2 experts independently seeding 3 batches with the same parameters (Figure S1G,H), and efficiency ranged from 27.6% to 80.2% (mean = 61.0%; Figure 1D), with 45.8% high and 8.3% low CM purity wells (Figure S1H). Notably, this differentiation contained more high purity outcomes (Figure S1H).

**FIGURE 2 |:**
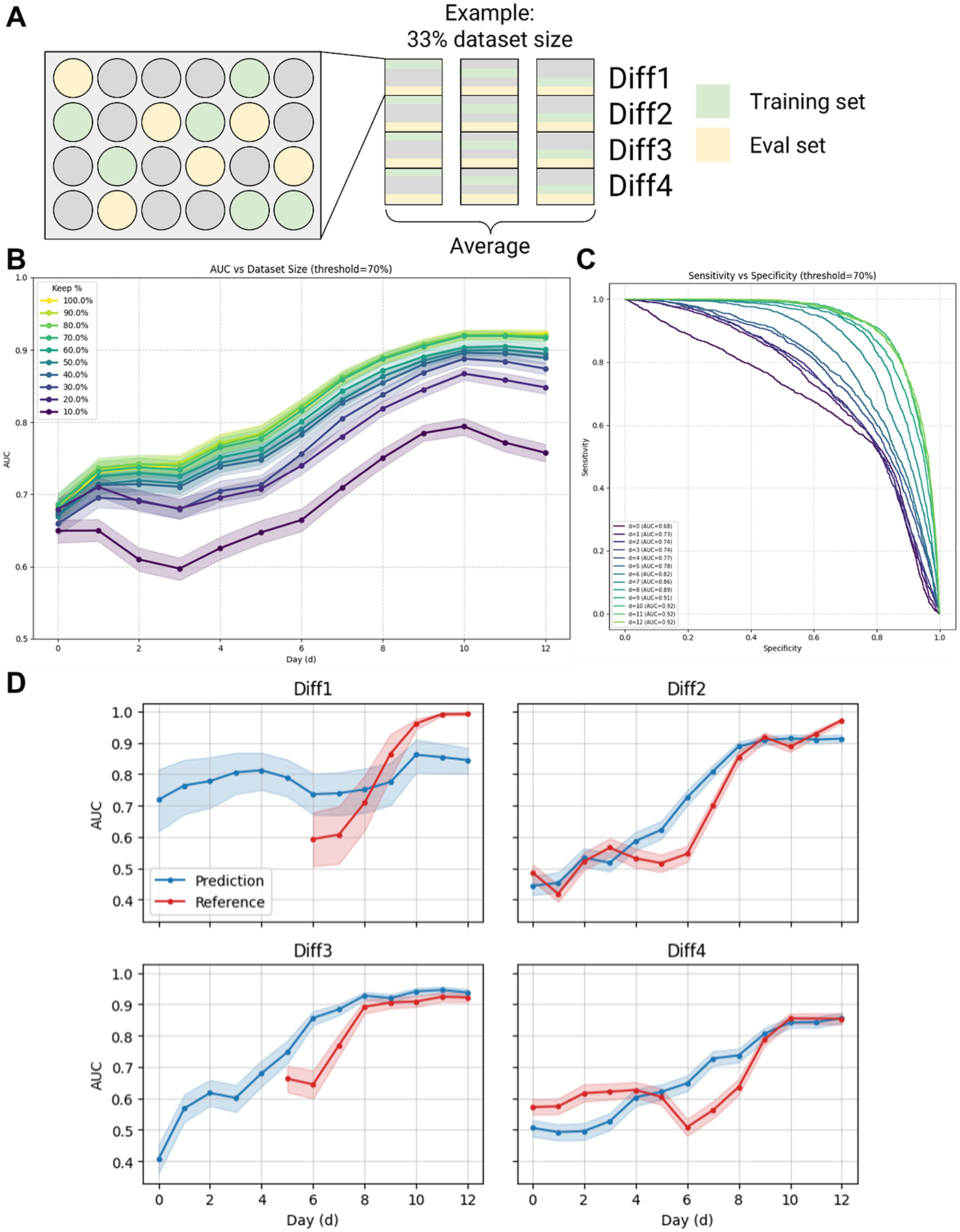
Phase contrast prediction model training across experiments with cross-validation over a random partition of holdout wells for a threshold of > 70% hPSC-CM differentiation efficiency (high purity differentiation). (A) Schematic of phase contrast prediction model cross-validation strategy for a random partition of holdout wells across hPSC-CM differentiations. All fields within a well were assigned to the same fold (training, evaluation, or holdout set). A data retention example (% Keep from (B)) of 33% is depicted. (B) Area under the curve (AUC) value from the receiver operating characteristic (ROC) curve over time across all hPSC-CM differentiations with different percentages of data retained in the model (Keep %). Bands indicate the lower and upper 2.5th percentiles of bootstrap resampled AUCs. (C) ROC curves (sensitivity vs. specificity) for 100% data retention (top) and 50% data retention (bottom) for each day of differentiation. (D) AUC comparison of the phase contrast prediction model (Prediction, blue) to the cTnT-GFP live-cell reporter (Reference, red) separated by differentiation experiment. Points represent mean field-level AUCs with lines depicting 95% CI.

To evaluate model performance in predicting hPSC-CM differentiation efficiency as high (> 70% CMs) or low (< 40% CMs) purity, we employed cross-validation over a random partition of wells and repeated this with random data subsets to assess the effect of dataset size (Figure 2A). For high efficiency predictions, the model with 100% data retention achieved an AUC > 0.70 by Day 1 and an AUC > 0.80 by Day 6 (Figure 2B,C). For low efficiency predictions, the model with 100% data retention performed better than the high efficiency prediction model with an AUC > 0.70 by Day 1 and an AUC > 0.80 by Day 4 (Figure S2A,B). These results demonstrate the ability to effectively predict high and low hPSC-CM differentiation efficiency early in differentiation, prior to CM emergence. As differentiation progressed, the ability to correctly predict end-point differentiation outcomes increased. After CM emergence, 100% data retention models identified high and low differentiation efficiency wells with an AUC > 0.90 by Day 9. To understand the relationship between the training dataset size and the ability to make accurate predictions, we performed a data ablation study. Model performance was statistically significantly reduced at all timepoints from Day 1 and beyond when 60% or less data was used for training to predict either high or low purity differentiations (*p* < 0.005, *N* = 1000, bootstrap resample method). This significant reduction in model performance was absent on nearly all days with 90% data retention (Figures 2B and S2A). These data suggest that increasing the number of replicates would not have substantially improved model performance for random well holdouts. Overall, these results demonstrate that phase contrast images contain predictive information and that our machine learning approach can effectively make reliable predictions based on this information.

To benchmark our phase contrast predictions, we compared the performance to live-cell cTnT-GFP reporter images, which should serve as a strong predictor of Day 12 cTnT measured by ICC at time points after cTnT expression begins. Despite the unfair comparison, across differentiations phase contrast predictions of high and low efficiency differentiation provided earlier information than a live-cell cTnT-GFP reporter and matched live-cell cTnT-GFP reporter performance from Days 8 to 12 (Figures 2D and S2C). However, the phase contrast model predictions performed differently across differentiations (Figures 2D and S2C). Notably, for predicting high efficiency differentiation in Differentiation 1, model predictions were high (AUCs > 0.70) throughout differentiation; yet, on Day 10, the live-cell cTnT-GFP reporter exhibited better performance than phase contrast model predictions. Phase contrast models performed best for Differentiations 2 and 3, with AUC values consistently higher than those of the live-cell cTnT-GFP reporter from Days 5 to 7. Similarly, for Differentiation 4, phase contrast model predictions were higher than the live-cell cTnT-GFP reporter from Days 6 to 8. The trends for low efficiency differentiation prediction across differentiations were similar to high efficiency differentiation predictions, with slightly better performance (Figure S2C). These results indicate the ability of phase contrast images to inform hPSC-CM differentiation efficiency early in differentiation, with differences across experiments likely due to differences in experiment design and differentiation outcomes.

**FIGURE 3 |:**
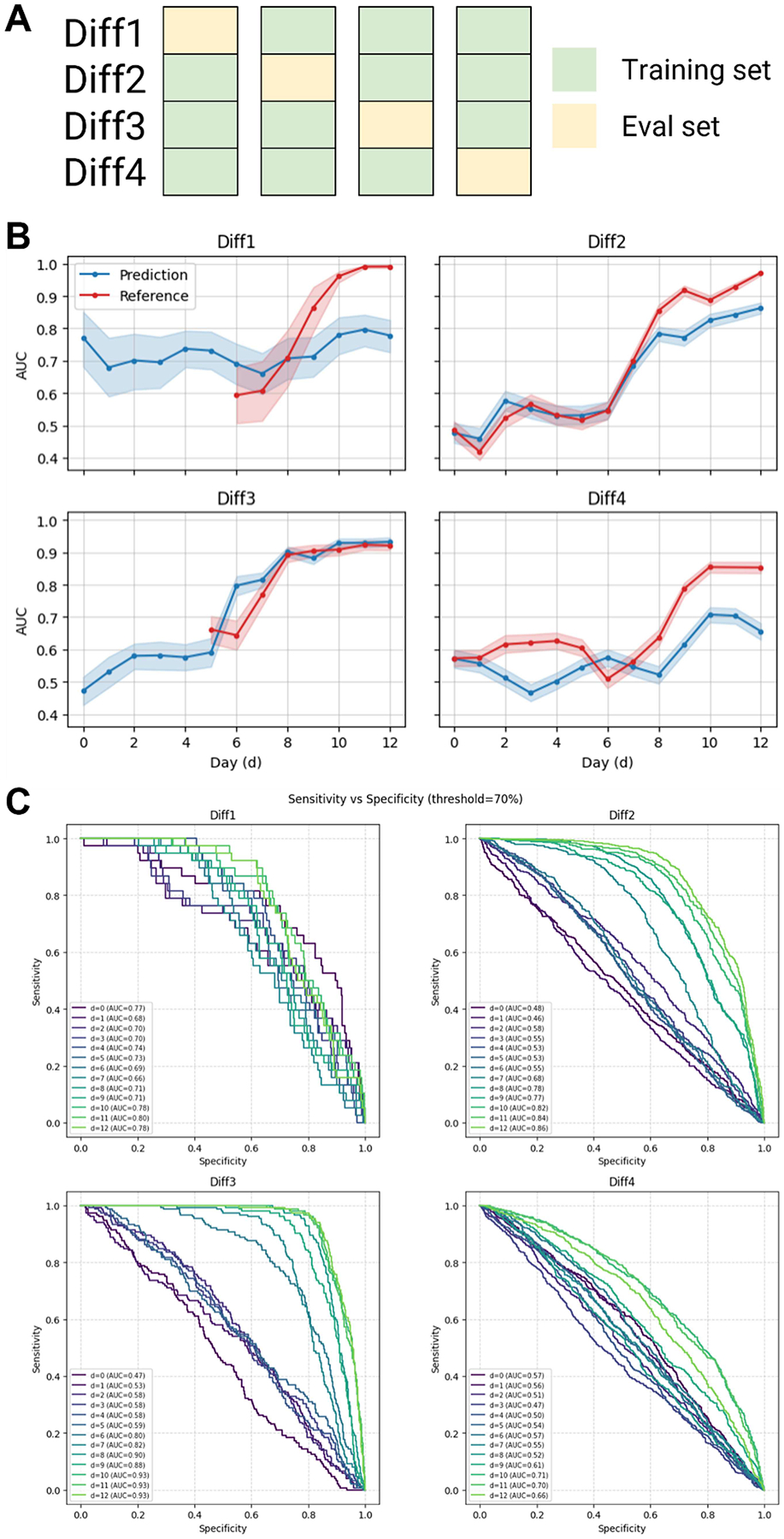
A generalized phase contrast prediction model evaluated on whole dataset holdouts matches the performance of a live-cell cTnT-GFP reporter for a threshold of > 70% hPSC-CM differentiation efficiency (high purity differentiation). (A) Schematic of phase contrast prediction model cross-validation strategy for whole dataset (differentiation experiment) holdouts. (B) AUC comparison for the phase contrast prediction model (Prediction, blue) to the cTnT-GFP live-cell reporter (Reference, red) separated by differentiation experiment. Points represent mean field-level AUCs with lines depicting 95% CI. (C) ROC curves (sensitivity vs. specificity) for the whole-dataset holdout phase contrast prediction models for each day of differentiation, separated by differentiation experiment.

In real-world model deployments, predictions must be made during ongoing differentiations for which no data from that run are included in the training set. Therefore, we trained models using phase contrast images from three of the four datasets and evaluated them on the holdout dataset in a round-robin fashion (Figure 3A). As expected, model performance for predicting high and low efficiency differentiation decreased with this holdout strategy compared to the random-well holdout strategy. Across differentiations, predictions achieved AUCs near 0.60 by Day 6 and AUCs near 0.70 from Days 10 to 12 (Figures 3B,C and S3A,B). Interestingly, model performance varied based on the specific training and holdout dataset splits.

For high efficiency prediction, phase contrast models performed similarly to cTnT-GFP live-cell reporter images for Differentiations 2 and 3, but performed worse for Differentiations 1 and 4 at most time points (Figure 3B). For Differentiation 1, the phase contrast model achieved an AUC above 0.80 by Day 11, demonstrating the ability to identify CMs (Figure 3C). Similarly, for Differentiation 2, the phase contrast model achieved an AUC above 0.80 by Day 10 (Figure 3C). The phase contrast model performed best for Differentiation 3, achieving an AUC above 0.80 by Day 7 (Figure 3C). For Differentiation 4, an AUC above 0.70 was achieved on Day 10 (Figure 3C). Phase contrast models showed comparable performance for low and high efficiency differentiation prediction, with similar AUC timing and variable agreement with cTnT-GFP reporters across differentiations (Figure S3A,B). Although model performance decreased under whole dataset cross-validation, predictions remained reliable and accurately identified CMs at later differentiation stages. The drop in model performance likely reflects dataset heterogeneity, and incorporating additional datasets to better capture outcome variability would likely improve phase contrast model performance under whole dataset cross-validation.

In this study, we illustrate that a random projection LDA machine learning model trained on phase-contrast images can predict hPSC-CM differentiation efficiency and accurately identify CMs. Using a random well holdout cross-validation strategy, we demonstrated accurate classification of high and low purity hPSC-CM differentiations as early as Day 1, prior to CM emergence. In comparison, CMs were not accurately identified using a cTnT-GFP live-cell reporter until Day 9. Model predictions remained acceptable using a dataset-level cross-validation strategy, which better reflects real-world deployment of these models, and in most cases matched the performance of the cTnT-GFP live-cell reporter, but relying only on phase contrast images.

As clinical applications of hPSC-derived cells expand, non-destructive differentiation monitoring will be increasingly critical for biomanufacturing. Phase contrast imaging is well-positioned for routine use due to its widespread availability and utility. Phase contrast imaging has been used to predict hPSC differentiation across all three germ layers, including CMs (Yang et al. 2023), skeletal myocytes (Hojo et al. 2025), hepatocytes (Marzec-Schmidt et al. 2023), and retinal pigment epithelial cells (Lien et al. 2023). Moreover, phase contrast imaging has been used to selectively ablate off-target cell types with laser purification during monolayer hPSC-CM differentiation (Yang et al. 2023). However, clinically relevant cell doses will likely require 3D differentiation, where CM identification has been demonstrated (Kandula et al. 2025; Mohammadi et al. 2023). Yet, phase contrast differentiation efficiency prediction and laser purification in 3D remain challenging. As machine learning integrates into CM manufacturing, our data demonstrate earlier prediction of differentiation outcome, enabling future efforts in protocol optimization, purification, prediction of therapeutic potency, and application to 3D.

## 3 | Materials and Methods

### 3.1 | hPSC Maintenance and Cardiomyocyte Differentiation

hPSCs were cultured in mTesR1 (STEMCELL Technologies 85850) and passaged with Versene (Life Technologies 15040066) for 6–10 min every 3–5 days at 60%–80% confluency. hPSCs were cultured on Matrigel (0.5 mg/plate or 11 μg/cm^2^, Corning 354263) in an incubator (Sanyo MCO-18AC, 37°C, 5% CO_2_, 95% RH) on six-well plates (Corning COSTAR 3516). The H9-cTnT-GFP hPSC line (WiCell, Madison, WI) was used in all experiments (Wrighton et al. 2014).

hPSCs were differentiated to CMs using the GiWi protocol (Lian et al. 2013). hPSCs were lifted with Accutase (Innovative Cell Technology AT104) and seeded on Day −2 at the indicated cell densities on ibidi 24-well plates (ibidi 82426) in 0.81 mL per well of mTesR1 with 5 μM Y-27632 (Tocris 1254). On Day −1, wells were fed with mTeSR1. On Day 0, differentiation was started in RPMI1640 (Life Technologies 11875119) with 1:50 v/v B27 minus insulin (B27−, Life Technologies 0050129SA) and CHIR99021 (5–12.5 μM, Selleckchem, S1263). On Day 1, wells were fed with B27−. On Day 3, wells were fed with 1:1 v/v B27− and conditioned medium with 5 μM IWP2 (Tocris 3533). On Day 5, wells were fed with B27 medium. On Days 7 and 10, wells were fed with RPMI1640 with 1:50 v/v B27 plus insulin (B27 +, Life Technologies 17504044).

### 3.2 | Phase Contrast, Fluorescent Reporter, and cTnT ICC Imaging

Phase contrast imaging was performed approximately daily from Days 0 to 12 on a Nikon Ti2-E microscope with an ORCA-Flash4.0 camera (Hamamatsu C13440–20CU). Intermittent, low-dose H9-cTnT-GFP reporter imaging was performed using 50% light intensity and 400 ms exposure with an Aura III light engine attachment (Lumencor 80–10306) utilizing 475/28 nm excitation. Whole-well imaging was achieved with 76 tiles (5% overlap) and framing inside the well.

On Day 12, wells were fixed for 30 min in 1% paraformaldehyde (Electron Microscopy Sciences 15710-S) in DPBS (ThermoFisher 14190144) and blocked with 1% (w/v) BSA (ThermoFisher BP1600) in DPBS. Wells were permeabilized and immunostained in 0.5% BSA with 0.1% Triton X-100 (ThermoFisher BP151) in DPBS using a cardiac troponin T primary antibody (msIgG1, ThermoFisher MA512960; 1:1000) overnight at 4°C, followed by goat anti-msIgG1 Alexa Fluor 647 secondary antibody (ThermoFisher 21240; 1:1000) for 1 h at room temperature in the dark. cTnT ICC binarization in NIS-Elements (v5.30.06) with experiment-specific thresholding was applied uniformly across wells. Imaging was at 10× magnification.

### 3.3 | Generation of Machine Learning Classification Models

Phase contrast images were aligned with binarized ICC images to generate consistent input image sequences and target % cTnT + area for each field of view (FOV). Each phase contrast image within a dataset was clipped and scaled to the global 1st and 99th percentile pixel intensities over all images within that dataset. For each image, 10 384 × 384 pixel crops were extracted and projected to a 512-dimensional vector using a randomly initialized ResNet-50 (He et al. 2016; torchvision v0.14). Random projection of pixels into an embedding vector is a simple approach for image prediction tasks that preserves strong performance without requiring complex pretraining, fine-tuning, or end-to-end training (Saxe et al. 2011). The crops were averaged for each FOV and reduced to 32 dimensions using principal components analysis. Missing timepoints were handled by forward-filling embeddings from the most recent prior timepoint. Target % cTnT+ area was classified into 10 mutually exclusive histogram bins based on global statistics for each training split. For each timepoint and FOV, input vectors were assembled by concatenating the 32-dimensional vectors from all images for that FOV up to that timepoint. For each timepoint, a multiclass LDA classifier was trained to predict the target histogram bin and yield a differentiation efficiency score by combining the predictions over each bin as an expected value. All software libraries used for analysis are open source, including Python (v3.10), NumPy (v1.23), pandas (v1.5), PyTorch (v1.13), torchvision (v0.14), and scikit-learn (v1.5).

### 3.4 | Statistical Analysis

Confidence intervals were computed via bootstrap resampling and represent the lower and upper 2.5th percentiles of resampled statistics. Figures for % cTnT+ area were created in GraphPad Prism (version 9.5.1). * for *p* < 0.05, ** for *p* < 0.01, *** for *p* < 0.001, and **** for *p* < 0.0001.

## Supplementary Material

Supplementary Information

Additional supporting information can be found online in the Supporting Information section.

**Figure S1:** Overview of hPSC-CM differentiation conditions including seeding density, CHIR99021 concentration, expert, and batch as well as differentiation efficiency outcomes per experiment.

**Figure S2:** Phase contrast prediction model training across experiments with cross-validation over a random partition of holdout wells for a threshold of <40% hPSC-CM differentiation efficiency (low purity differentiation).

**Figure S3:** A generalized phase contrast prediction model evaluated on whole dataset holdouts matches the performance of a live-cell cTnT-GFP reporter for a threshold of <40% hPSC-CM differentiation efficiency (low purity differentiation).

## Data Availability

The data that support the findings of this study are available from the corresponding author upon reasonable request.
