# Supplementary material for: Machine Learning Models of Phase Contrast Images Predict Efficiency of Human Pluripotent Stem Cell Differentiation to Cardiomyocytes
Source: Biotechnol Bioeng. Author manuscript; Available in PMC 2026 Jun 16. (PMC13270429; doi:10.1002/bit.70241)
Supplement: Supplementary Information [file NIHMS2184372-supplement-Supplementary_Information.docx]

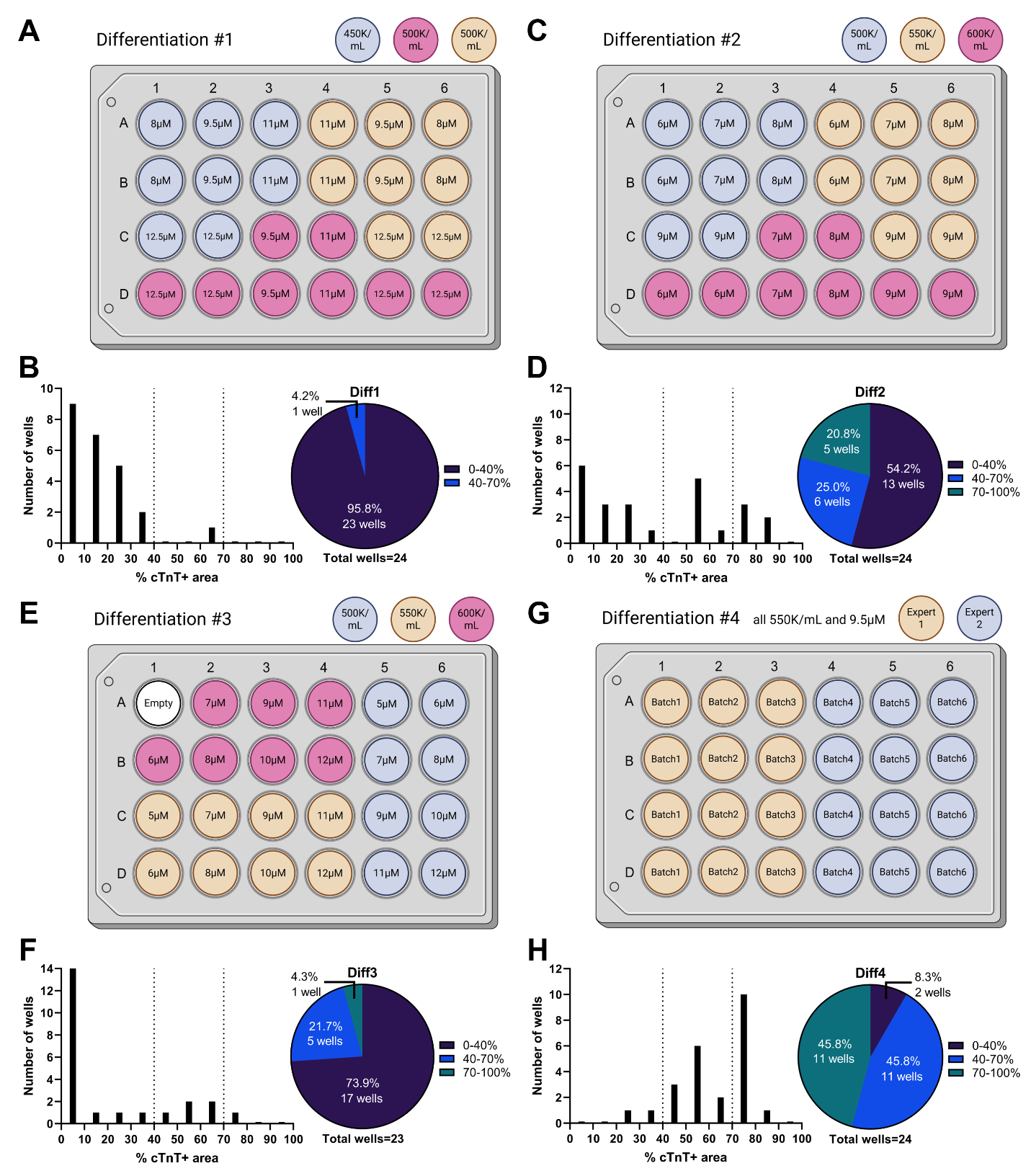


**Figure S1. Overview of hPSC-CM differentiation conditions including seeding density, CHIR99021 concentration, expert, and batch as well as differentiation efficiency outcomes per experiment. A)** Differentiation 1 plate design and experimental conditions for seeding density (450, 500, 550K cells/mL) and CHIR99021 (8, 9.5, 11, 12.5 µM) concentration. N=2 wells per condition. **B)** Differentiation 1 histogram (left) for hPSC-CM differentiation efficiency (percentage of cTnT+ cells by area). N=24 wells. Vertical dotted lines at 40% and 70% cTnT+ cells indicate thresholds for low and high efficiency CM differentiation respectively. Differentiation 1 pie chart (right) for high (>70% cTnT+ cells), low (<40% cTnT+ cells), and intermediate (40-70% cTnT+ cells) differentiation efficiency bins. **C)** Differentiation 2 plate design and experimental conditions for seeding density (500, 550, 600 K cells/mL) and CHIR99021 (6, 7, 8, 9 µM) concentration. N=2 wells per condition. **D)** Differentiation 2 histogram (left) for hPSC-CM differentiation efficiency (percentage of cTnT+ cells by area). N=24 wells. Vertical dotted lines at 40% and 70% cTnT+ cells indicate thresholds for low and high efficiency CM differentiation respectively. Differentiation 2 pie chart (right) for high (>70% cTnT+ cells), low (<40% cTnT+ cells), and intermediate (40-70% cTnT+ cells) differentiation efficiency bins. **E)** Differentiation 3 plate design and experimental conditions for seeding density (500, 550, 600 K cells/mL) and CHIR99021 (5, 6, 7, 8, 9, 10, 11, 12 µM) concentration. N=1 well per condition (600K cells/mL and 5 µM condition omitted). **F)** Differentiation 3 histogram (left) for hPSC-CM differentiation efficiency (percentage of cTnT+ cells by area). N=23 wells. Vertical dotted lines at 40% and 70% cTnT+ cells indicate thresholds for low and high efficiency CM differentiation respectively. Differentiation 3 pie chart (right) for high (>70% cTnT+ cells), low (<40% cTnT+ cells), and intermediate (40-70% cTnT+ cells) differentiation efficiency bins. **G)** Differentiation 4 plate design and experimental conditions for expert (1, 2) and differentiation batch (3 per expert). Separate batches were lifted, counted, and seeded for differentiation independently. N=4 wells per condition. **H)** Differentiation 4 histogram (left) for hPSC-CM differentiation efficiency (percentage of cTnT+ cells by area). N=24 wells. Vertical dotted lines at 40% and 70% cTnT+ cells indicate thresholds for low and high efficiency CM differentiation respectively. Differentiation 4 pie chart (right) for high (>70% cTnT+ cells), low (<40% cTnT+ cells), and intermediate (40-70% cTnT+ cells) differentiation efficiency bins.

**
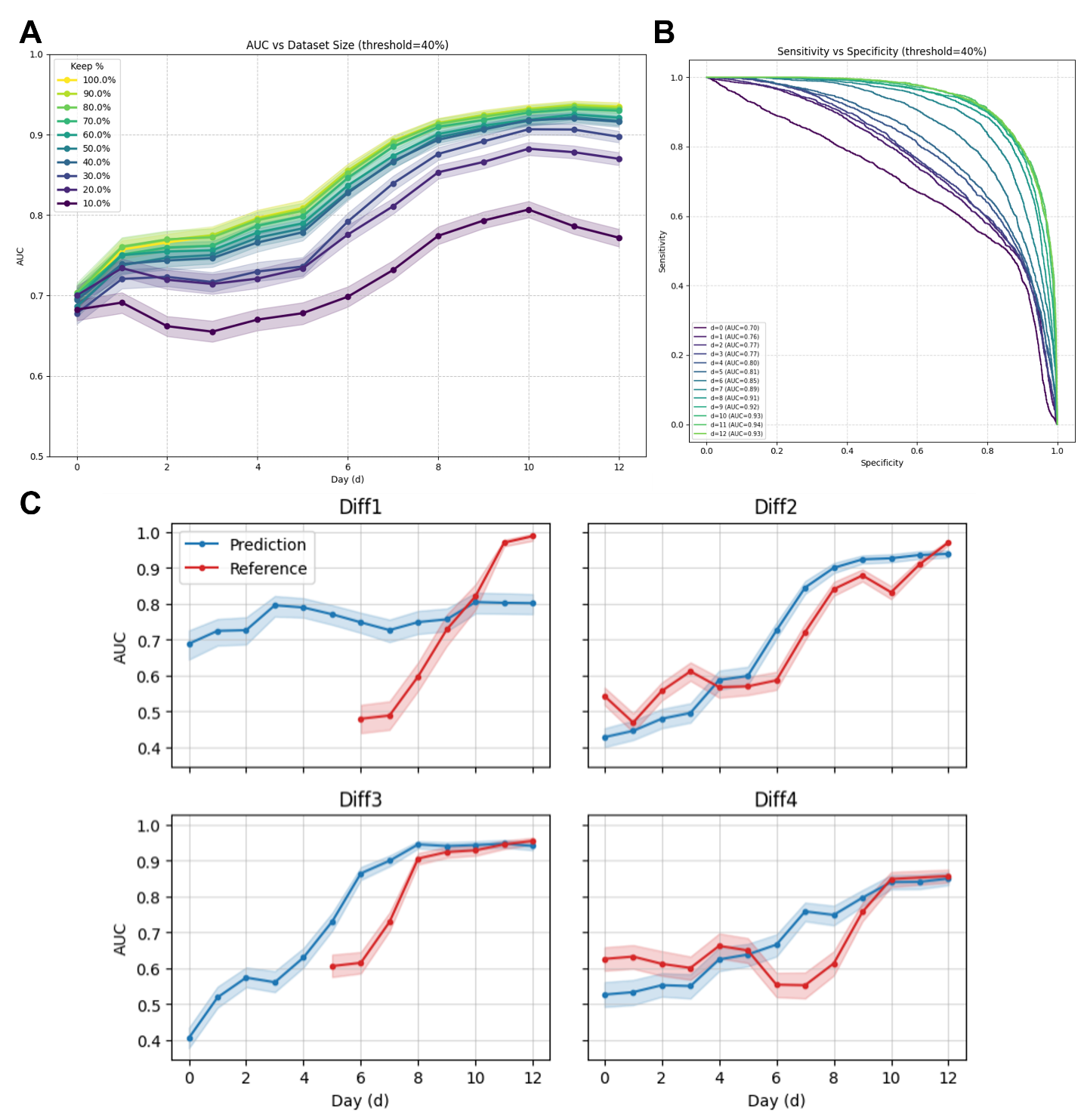
**

**Figure S2. Phase contrast prediction model training across experiments with cross-validation over a random partition of holdout wells for a threshold of <40% hPSC-CM differentiation efficiency (low purity differentiation).** **A)** Area under the curve (AUC) value from the receiver operating characteristic (ROC) curve over time across all hPSC-CM differentiations with different percentages of data retained in the model (Keep %). **B)** ROC curves (sensitivity versus specificity) for 100% data retention (top) and 50% data retention (bottom) for each day of differentiation. **C)** AUC comparison of the phase contrast prediction model (Prediction, blue) to the cTnT-GFP live-cell reporter (Reference, red). **D)** AUC comparison of the phase contrast prediction model (Prediction, blue) to the cTnT-GFP live-cell reporter (Baseline, red) separated by differentiation experiment. Points represent mean field-level AUCs with lines depicting 95% CI.

**
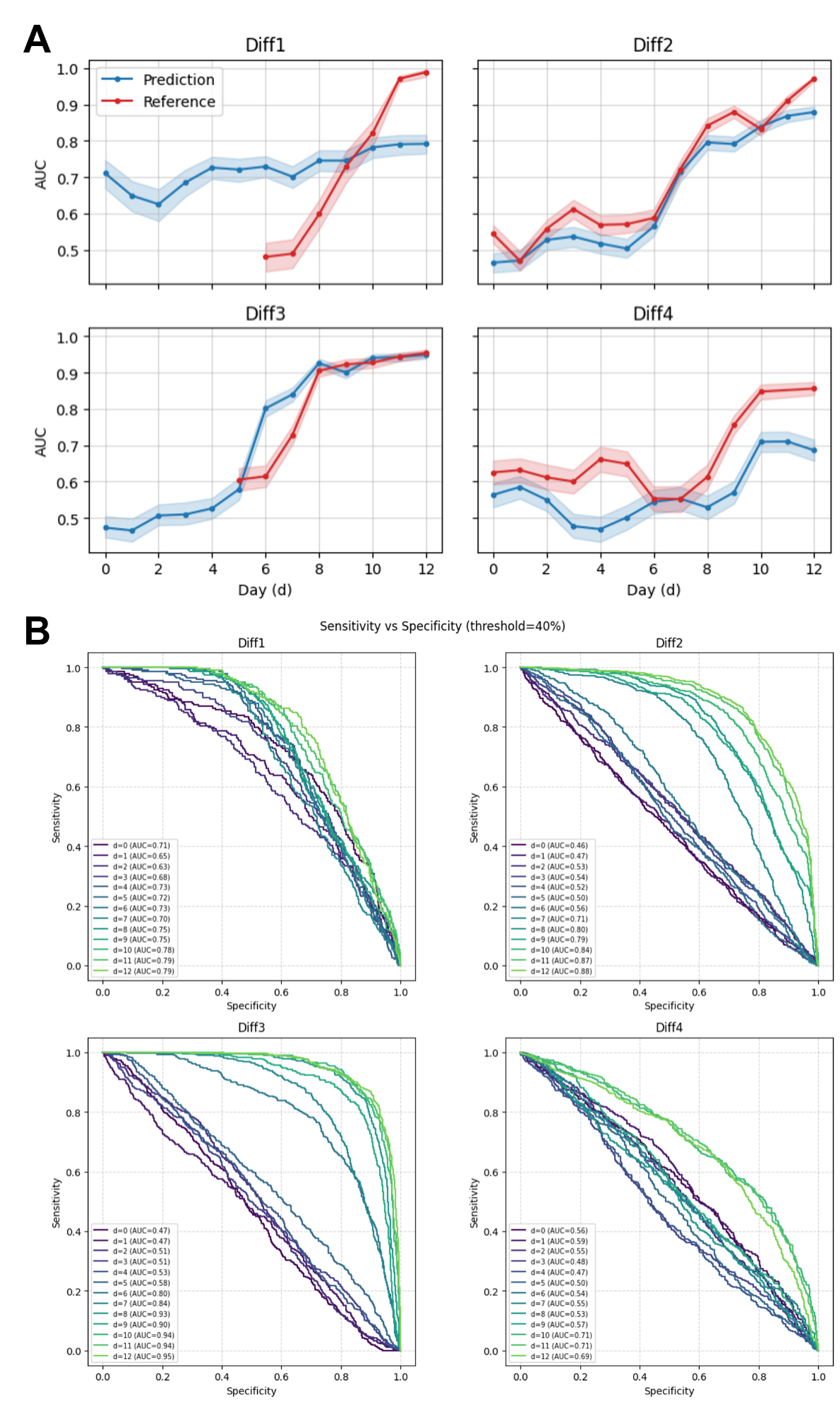
**

**Figure S3. A generalized phase contrast prediction model evaluated on whole dataset holdouts matches the performance of a live-cell cTnT-GFP reporter for a threshold of <40% hPSC-CM differentiation efficiency (low purity differentiation). A)** AUC comparison for the phase contrast prediction model (Prediction, blue) to the cTnT-GFP live-cell reporter (Baseline, red) separated by differentiation experiment. Points represent mean field-level AUCs with lines depicting 95% CI. **B)** ROC curves (sensitivity versus specificity) for whole dataset holdout phase contrast prediction models for each day of differentiation separated by differentiation experiment.
